# Serum Albumin, but not Bilirubin, is Associated with Diabetic Chronic Vascular Complications in a Chinese Type 2 Diabetic Population

**DOI:** 10.1038/s41598-019-48486-6

**Published:** 2019-08-19

**Authors:** Yu Zhu, Xiaoling Cai, Yan Liu, Mengdie Hu, Lingli Zhou, Wei Liu, Jing Wu, Rui Zhang, Xueying Gao, Wenjia Yang, Simin Zhang, Siqian Gong, Yingying Luo, Meng Li, Leili Gao, Ling Chen, Jing Chen, Xiuting Huang, Qian Ren, Xiuying Zhang, Xianghai Zhou, Xueyao Han, Linong Ji

**Affiliations:** Department of Endocrinology and Metabolism, Peking University People’s Hospital, Peking University Diabetes Centre, Beijing, 100044 China

**Keywords:** Health sciences, Health sciences, Health sciences, Health sciences

## Abstract

To identify the factors associated with serum total bilirubin (STB) and determine whether STB is independently associated with diabetic retinopathy (DR) or diabetic kidney disease (DKD), 1,665 Chinese patients with type 2 diabetes (T2DM) (248 outpatients newly diagnosed with T2DM [NDM] and 1,417 inpatients previously diagnosed with T2DM [PDM]) were studied. Clinical and biochemical information was collected, and a single nucleotide polymorphism (rs6704078) of the *UGT1A1* gene was genotyped in 1,059 individuals. Multiple linear regression showed that STB was associated with haemoglobin concentration, platelet count, and serum triglyceride concentration in NDM and PDM patients, and with serum albumin, duration of diabetes, and smoking in PDM patients. In patients with PDM, multiple logistic regression revealed that serum albumin was associated with DR (odds ratio [OR] = 0.92, 95% confidence interval [CI]: 0.87–0.96, *p* = 0.001) and DKD (OR = 0.93, 95% CI: 0.88–0.98, *p* = 0.005) after adjustment for STB, STB-related factors, and risk factors for DR and DKD. In addition, patients with the T allele of rs6704078 had higher STB (13.2 [10.4–17.9] μmol/L *versus* 11.8 (9.4–14.8) μmol/L; *p* < 0.001) and similar risks of DR or DKD to those without the T allele. Thus, serum albumin, but not STB, is associated with DR and DKD.

## Introduction

During recent decades, accumulating evidence has indicated that both oxidative stress and chronic inflammation are important in the development of type 2 diabetes (T2DM) and its chronic complications, including diabetic retinopathy (DR) and diabetic kidney disease (DKD)^1–3^. The total serum concentration of bilirubin, a physiologic antioxidant, has recently been reported to be associated with the risks of cardiovascular disease, diabetes and diabetic chronic vascular complications in a number of cross-sectional and observational studies^4–11^, but Mendelian randomization studies have not supported a causal relationship between STB, and stroke and coronary heart disease (CHD)^12–14^. Therefore, it is likely that unknown or unquantifiable confounders explain the association of serum total bilirubin (STB) with vascular complications observed in the previous studies, rather than STB itself.

Perhaps significantly, in addition to moderate hyperbilirubinemia, higher haemoglobin concentrations and low platelet counts characterize patients with Gilbert syndrome^15^, and patients with this syndrome have also been reported to have low risks of cardiovascular disease and diabetes^16^. It remains to be determined whether the relationship between STB and cardiovascular disease depends on haemoglobin concentration or platelet count in this syndrome. However, some case-control studies have shown that low haemoglobin concentration is associated with a higher risk of diabetic retinopathy^17^, high platelet count and activity are well-known risk factors for cardiovascular disease, and anti-platelet drugs have been widely used for the prevention of cardiovascular disease. Therefore, it is necessary to adjust for these confounders when evaluating the relationship between STB and diabetic vascular disease.

However, the significance of STB-related factors has not been established in patients with T2DM, and in all the previous studies of the relationship between STB and vascular diseases, a number of the potential confounders, such as haemoglobin concentration and platelet count, have not been adjusted for. Therefore, we aimed to evaluate the relationships between STB and the prevalences of DR and DKD, while accounting for the effects of haemoglobin concentration, platelet count, and other blood parameters, in a Chinese population with T2DM.

## Methods

### Ethics statement

This study was performed in accordance with the tenets of the Declaration of Helsinki and approved by the ethics committee of Peking University People’s Hospital (authorization numbers 2013-12 and 2010-71). Written informed consent was obtained from all the participants.

### Study population

#### Patients with newly diagnosed T2DM (NDM)

Between August 2014 and May 2018, a total of 248 patients with NDM were consecutively recruited in the Outpatient Clinic of the Department of Endocrinology and Metabolism, Peking University People’s Hospital. These patients had never previously been treated using antidiabetic drugs or lifestyle interventions. This sample was recruited to determine the associations between STB and other blood parameters, to avoid the confounding effects of the use of hypoglycaemic agents and the duration of diabetes. Blood and urine samples were obtained before these patients began treatment for diabetes. Their clinical characteristics are summarized in Table 1.

**Table 1:** Clinical characteristics of outpatients with newly diagnosed type 2 diabetes (NDM) and inpatients with previously diagnosed type 2 diabetes (PDM).

Characteristic	The clinical characteristics	
	NDM	PDM
	(n = 248)	(n = 1417)
Men/women, n	150/98	923/494
Age, years	49 ± 11	54 ± 13
Duration, years	—	10 (4–15)
BMI, kg/m^2^	27.5 ± 4.0	26.1 ± 3.9
WC for female, cm	91 ± 9	93 ± 11
WC for male, cm	96 ± 11	96 ± 11
SBP, mmHg	128 ± 16	130 ± 17
DBP, mmHg	81 ± 9	79 ± 11
FPG, mmol/l	8.5 (7.2–11.3)	7.2 (5.8–8.9)
HbA1c, %	9.0 ± 2.2	9.3 ± 2.4
TC, mmol/l	5.2 ± 1.2	4.5 ± 1.1
TG, mmol/l	1.9 (1.3–2.7)	1.6 (1.1–2.4)
HDL-c for female, mmol/l	1.15 (1.04–1.37)	1.05 (0.90–1.21)
HDL-c for male, mmol/l	1.03 (0.91–1.17)	0.90 (0.78–1.04)
LDL-c, mmol/l	3.0 ± 0.9	2.5 ± 0.8
ALT, u/l	29 (20–43)	19 (14–30)
AST, u/l	22 (17–29)	18 (15–24)
ALB, g/l	46.0 ± 2.7	40.5 ± 3.2
T-BIL, μmol/l	12.7 (10.3–16.2)	11.7 (9.2–14.6)
CRE, μmol/l	64 (53–75)	64 (54–74)
UA for female, μmol/l	316.0 (261.0–358.0)	308.0 (261.0–369.0)
UA for male, μmol/l	351.0 (309.8–421.8)	363.0 (306.0–425.8)
High-sensitivity CRP, mg/l	2.4 (1.3–4.5)	—
UACR, mg/g	10.1 (5.2–22.4)	6.6 (2.9–21.8)
WBC, 10^9^/l	7.16 ± 2.2	6.6 ± 1.6
RBC, 10^12^/l	5.1 ± 0.5	4.5 ± 0.5
Hb, g/dl	15.1 ± 1.4	13.8 ± 1.3
PLT, 10^9^/dl	24.4 ± 5.9	19.9 ± 5.0
Smoking, n (%)	84 (33.9)	639 (45.1)
Hypertension, n (%)	90 (36.3)	720 (50.8)
Dyslipidemia, n (%)	172 (69.4)	1011 (71.3)
CHD, n (%)	10 (4.0)	196 (13.8)
CVD, n (%)	5 (2.0)	178 (12.6)
PAD, n (%)	115 (45.6)	1221 (86.2)
DR, n (%)	16 (6.5)	297 (20.9)
DKD, n (%)	45 (18.1)	256 (18.2)

Values are mean ± SD, median (interquartile range), or n (%).

WC, waist circumference; SBP, systolic blood pressure; DBP, diastolic blood pressure; ALB, serum albumin; T-BIL, total bilirubin; CRE, serum creatinine; UA, uric acid; TC, total cholesterol; TG, triglycerides; HDL-c, high-density lipoprotein-cholesterol; LDL-c, low-density lipoprotein-cholesterol; ALT, alanine transaminase; AST, aspartate aminotransferase; UACR, urine albumin-creatinine ratio; WBC, white blood cell count; RBC, red blood cell count; Hb, haemoglobin concentration; PLT, platelet count; CHD, coronary heart disease; PAD, peripheral arterial disease; CVD, cerebral vascular disease; DR, diabetic retinopathy; DKD, diabetic kidney disease.

#### Patients with previously diagnosed T2DM (PDM)

A total of 1,417 consecutive inpatients with T2DM who were hospitalized at the Department of Endocrinology and Metabolism, Peking University People’s Hospital, between 2011 and 2017, were enrolled in this study. All the patients had been taking hypoglycaemic agents before hospitalization. This sample was used to validate the findings obtained in the NDM population and assess the relationships of STB and related parameters with diabetic chronic vascular complications. Their clinical characteristics are summarized in Table 1.

### Inclusion and exclusion criteria

All the participants were of Chinese Han origin and had been diagnosed with T2DM by diabetologists. Participants with a fasting plasma glucose (FPG) below 7 mmol/l and glycated haemoglobin (HbA1c) below 6.5% undertook a 75-g oral glucose tolerance test (OGTT). Diabetes was diagnosed according to World Health Organization criteria, on the basis of an FPG ≥7.0 mmol/L, a 2-h plasma glucose during the OGTT ≥11.1 mmol/L, and/or HbA1c ≥6.5%. Individuals were excluded if they had typical clinical features of type 1 diabetes (severe insulin deficiency [fasting serum C peptide <0.1 ng/ml] or dependency on insulin treatment within 2 years of a diagnosis of diabetes); had another specific form of diabetes, such as diabetes secondary to chronic pancreatitis or steroid treatment; were positive for glutamic acid decarboxylase, islet cell, and insulin autoantibody; had abnormal red or white blood cells in their urine, or other kidney disease, such as urolithiasis; had cancer, infection, acute diabetic complications, autoimmune disease, a blood disease, or obstructive jaundice; had high serum alanine transaminase (ALT) activity (≥twice the upper limit of the normal range; had a white blood cell count <3.5 × 10^9^/L, haemoglobin concentration <110 g/l in women and <120 g/l in men, or platelet count <10 × 10^9^/L; or had a severe chronic liver disease, such as cirrhosis, viral hepatitis, or chronic alcohol-induced liver dysfunction.

### Biochemical measurements and clinical information

The sex, age, duration of diabetes, smoking history, and presence of comorbidities or complications were recorded for each participant. The participants’ height, body mass, body mass index (BMI), waist circumference, systolic blood pressure (SBP) and diastolic blood pressure (DBP) were measured.

After an overnight fast of 8–10 h, venous blood was collected for the measurement of FPG, total cholesterol, high-density lipoprotein-cholesterol (HDL-c), low-density lipoprotein-cholesterol (LDL-c), triglycerides (TG), creatinine, uric acid, STB, albumin, high-sensitivity C-reactive protein (only for NDM patients), and alanine transaminase and aspartate aminotransferase activities, using a Hitachi LST008 analyser. HbA1c was measured using high-performance liquid chromatography (Ultra2 HbA1c Detector, Primus Corporation, Atlanta, GA, USA). Roche C311 was used to measure the urinary albumin/creatinine ratio (UACR). White blood cell count, red blood cell count, haemoglobin concentration and platelet count were measured using a haematology analyser.

In our study, DKD was defined by a UACR ≥30 mg/g and/or renal insufficiency. A diagnosis of DR was made on the basis of direct ophthalmoscopy, fundic camera (TRC-NW100) examination or a history of laser photocoagulation therapy. CHD was diagnosed using the patients’ medical history (presence of angina or myocardial infarction and the results of computed tomography or coronary angiography). Cerebrovascular disease (CVD) was defined by a history of transient ischaemic attack, or ischaemic or haemorrhagic stroke. Hypertension was defined by a SBP ≥140 mmHg, a DBP ≥90 mmHg and/or current treatment for hypertension. Dyslipidaemia was defined by a total cholesterol ≥5.18 mmol/l, triglycerides ≥1.70 mmol/l, LDL-c ≥3.37 mmol/l, HDL-c <1.3 mmol/l in women or HDL-c <1.04 mmol/l in men, or treatment with anti-hyperlipidaemic agents^18^. Peripheral arterial disease (PAD) was identified using ultrasonography; if plaques were detected in the carotid artery or the arteries of the lower extremities, a diagnosis of peripheral vascular atherosclerosis was made.

Blood and urinary biochemical data, and diagnoses of PAD, DR, DKD, hypertension or dyslipidaemia were collected from the participants’ medical records, and the presence of CHD or CVD and smoking status were self-reported.

### Study of the association between the UDP-glucuronyl transferase (UGT1A1) gene polymorphism and fasting STB

DNA samples obtained from whole blood were available from 1,059 patients with T2DM, because some patients did not agree to provide samples or their samples had been exhausted. The rs6742078 single nucleotide polymorphism (SNP), which has previously been reported to be associated with STB and to explain more of the variation in STB than other SNPs^8,12–14^, was genotyped by DNA sequencing or using a TaqMan SNP Genotyping Assay (Applied Biosystems, Foster City, CA, USA). For quality control, 5% of the samples were re-genotyped in a blinded fashion by DNA sequencing.

### Statistical analysis

Statistical analysis was performed using SPSS for Windows, v. 23.0 (IBM, Inc., Armonk, NY, USA). Normally distributed continuous data are presented as means and standard deviations (±SD) and non-normally distributed data are presented as medians (inter quartile range, IQR). Categorical variables are presented as numbers and percentages. The duration of diabetes, serum HDL-c, triglycerides, STB, creatinine, UACR, urine acid, alanine transaminase, aspartate aminotransferase and FPG were subjected to natural logarithmic transformation to obtain normally distributed data, prior to statistical analysis. Independent *t*-tests were used to compare the means of quantitative traits and the chi-square test was used for qualitative traits. Multiple linear regression was performed with ln-transformed STB as the dependent variable, and sex, age, BMI, waist circumference, FPG, HbA1c, SBP, DBP, STB, serum albumin, triglycerides, HDL-c, LDL-c, serum creatinine, UACR, white blood cell count, haemoglobin concentration, platelet count, and smoking status as independent variables. A logistic regression analysis with a forward method was also performed with DR or DKD as the dependent variable, and sex, age, BMI, waist circumference, FPG, HbA1c, SBP, DBP, STB, serum albumin, triglycerides, HDL-c, LDL-c, white blood cell count, haemoglobin concentration, platelet count, smoking, hypertension and dyslipidaemia as independent variables. *P* < 0.05 was considered to represent statistical significance.

## Results

### Relationships between serum bilirubin and other blood and urine parameters in patients with NDM or PDM

In the participants with NDM, in whom there was no confounding by the use hypoglycaemic agents or the duration of diabetes, multiple linear regression analysis was conducted with ln-transformed STB as the dependent variable, and sex, age, BMI, waist circumference, FPG, HbA1c, SBP, DBP, serum albumin, alanine transaminase, triglycerides, HDL-c, LDL-c, creatinine, UACR, high sensitivity C-reactive protein, white blood cell count, haemoglobin concentration, platelet count and smoking status as independent variables. This showed that haemoglobin concentration (standardized beta coefficient = 0.24, *p* = 0.01), platelet count (standardized beta coefficient = −0.18, *p* = 0.01) and triglycerides (standardized beta coefficient = −0.25, *p* = 0.001) were independently associated with STB.

In the patients with PDM, multiple linear regression analysis was conducted with ln-transformed STB as the dependent variable, and sex, age, BMI, waist circumference, FPG, HbA1c, SBP, DBP, serum albumin, alanine transaminase, triglycerides, HDL-c, LDL-c, creatinine, UACR, white blood cell count, haemoglobin concentration, platelet count and smoking status as independent variables. This showed that haemoglobin concentration (standardized beta coefficient = 0.25, *p* < 0.001), platelet count (standardized beta coefficient = −0.12, *p* < 0.001), serum albumin (standardized beta coefficient = 0.13, *p* < 0.001), triglycerides (standardized beta coefficient = −0.20, *p* < 0.001), duration of diabetes (standardized beta coefficient = −0.10, *p* = 0.001) and smoking status (standardized beta coefficient = −0.08, *p* = 0.009) were independently associated with STB.

### Relationships of STB and related parameters with diabetic chronic vascular complications

The associations of STB and related factors with diabetic chronic vascular complications were evaluated in patients with PDM. Multiple logistic regression analysis (Table 2) was conducted with DR or DKD as the dependent variable, and sex, age, duration of diabetes, BMI, waist circumference, FPG, HbA1c, SBP, DBP, serum albumin, alanine transaminase, STB, triglycerides, HDL-c, LDL-c, white blood cell count, haemoglobin concentration, platelet count and smoking status as independent variables. The results showed that age, duration of diabetes, FPG, serum albumin and SBP were independently associated with DR, and that age, duration of diabetes, FPG, serum albumin, triglycerides, SBP, hypertension and white blood cell count were independently associated with DKD. After excluding 30 patients who had UACR >1,000 mg/g, the associations between serum albumin and DKD or DR remained significant.

**Table 2:** Multiple logistic regression analysis of the relationships between diabetic retinopathy or diabetes kidney disease and serum bilirubin in inpatients with previously diagnosed type 2 diabetes (PDM).

	DR			DKD		
	B	OR (95% CI)	P	B	OR (95% CI)	P
Female	0.017	1.02(0.65–1.59)	0.940	−0.482	0.62(0.38–1.01)	0.057
Age, years	−0.031	0.97(0.95–0.97)	<0.001	−0.02	0.96(0.96–0.99)	0.033
Duration, years	0.092	1.10(1.07–1.12)	<0.001	0.073	1.08(1.05–1.10)	<0.001
BMI, kg/m^2^	−0.052	0.95(0.88–1.02)	0.156	0.012	1.01(0.94–1.09)	0.761
WC, cm	0.021	1.02(0.99–1.05)	0.116	0.011	1.01(0.98–1.04)	0.420
ALT, u/l	−0.007	0.99(0.93–1.01)	0.181	−0.010	0.99(0.98–1.01)	0.088
FPG, mmol/l	0.091	1.09(1.03–1.16)	0.003	0.082	1.09(1.01–1.16)	0.019
HbA1c, %	0.033	1.03(0.97–1.10)	0.295	−0.002	0.99(0.92–1.08)	0.966
Hb, g/dl	−0.129	0.88(0.76–1.02)	0.090	−0.147	0.86(0.74–1.01)	0.070
Alb, g/l	−0.086	0.92(0.87–0.96)	0.001	−0.075	0.93(0.88–0.98)	0.005
PLT, 10^9^/dl	−0.027	0.97(0.94–1.01)	0.115	−0.024	0.97(0.94–1.01)	0.191
T-Bil, μmol/l	−0.023	0.98(0.94–1.01)	0.202	−0.016	0.98(0.95–1.02)	0.398
WBC, 10^9^/l	0.058	1.06(0.96–1.17)	0.243	0.238	1.27(1.15–1.40)	<0.001
TG, mmol/l	−0.017	0.98(0.92–1.06)	0.640	0.066	1.07(1.01–1.14)	0.035
LDL-c, mmol/l	0.051	1.05(0.87–1.27)	0.595	0.014	1.01(0.83–1.24)	0.894
HDL-c, mmol/l	−0.321	0.73(0.42–1.24)	0.243	0.206	1.23(0.84–1.80)	0.294
SBP, mmHg	0.019	1.02(1.01–1.03)	0.002	0.019	1.02(1.01–1.03)	0.003
DBP, mmHg	−0.014	0.99(0.97–1.01)	0.119	−0.006	0.99(0.98–1.01)	0.508
Hypertension	0.246	1.28(0.90–1.81)	0.164	0.960	2.61(1.78–3.84)	<0.001
Dislipidemia	0.024	1.02(0.74–1.43)	0.888	−0.088	0.92(0.64–1.32)	0.634
Smoking	0.02	0.92(0.71–1.48)	0.92	0.136	1.15(0.77–1.70)	0.497

WC, waist circumference; SBP, systolic blood pressure; DBP, diastolic blood pressure; FPG, fasting plasma glucose; ALB, serum albumin; T-BIL, total bilirubin; WBC, white blood cell count; RBC, red blood cell count; Hb, haemoglobin concentration; PLT, platelet count; TG, triglycerides; HDL-c, high-density lipoprotein-cholesterol; LDL-c: low-density lipoprotein-cholesterol; ALT, alanine transaminase.

### The relationship between the UGT1A1 gene polymorphism and fasting STB

A total of 1,059 participants with NDM or PDM were genotyped for rs6742078. The frequencies of the three genotypes (GG, GT and TT) were 75.5%, 21.0% and 1.5%, respectively. The frequencies of the G and T alleles were 88.0% and 12.0%, respectively, which are very similar to those (89% and 11%) recorded for the Chinese Han population in the 1,000-genome database (http://phase3browser.1000genomes.org/index.html). The genotype distribution was in Hardy-Weinberg equilibrium (calculated using software at http://www.oege.org/software/hwe-mr-calc.html). As shown in Table 3, the STB in patients with the GG genotype was lower than that of those with GT or TT genotypes. Multiple linear regression analysis showed that STB was associated with the T allele under the dominant model, after adjustment for sex, age, BMI, waist circumference, FPG, HbA1c, SBP, DBP, serum albumin, alanine transaminase, triglycerides, HDL-c, LDL-c, creatinine, UACR, white blood cell count, haemoglobin concentration, platelet count and smoking status. However, the prevalence of DR, DKD, CHD, CVD, PVD, hypertension and dyslipidaemia were similar in participants with the GG and GT/TT genotypes. The T allele was not associated with DR (odds ratio [OR] = 0.94, 95% confidence interval [CI]: 0.68–1.31, *p* = 0.72) or DKD (OR = 0.87, 95% CI: 0.62–1.23, *p* = 0.44) in logistic regression analysis.

**Table 3:** Clinical characteristics of patients with differing *UGT1A1* genotype.

	GG	GT/TT	p
	(n = 821)	(n = 238)	
Men/women, n	494/327	144/94	0.93
Age, years	55 ± 12	55 ± 15	0.67
BMI, kg/m^2^	26.7 ± 4.4	26.5 ± 4.2	0.69
Duration, years	6(0.1–13)	5(0.1–14)	0.75
WC for female, cm	92.9 ± 10.1	94.6 ± 10.2	0.16
WC for male, cm	96.6 ± 10.2	94.4 ± 13.5	0.08
SBP, mmHg	131 ± 18	133 ± 17	0.15
DBP, mmHg	79 ± 11	80 ± 11	0.76
FPG, mmol/l	7.4(6.1–9.5)	7.5(5.9–9.5).	0.76
HbA1c, %	8.6(7.3–10.4)	8.8(7.5–11.2)	0.06
TC, mmol/l	4.6 ± 1.1	4.7 ± 1.1	0.57
TG, mmol/l	2.2(1.4–3.2)	2.1(1.4–3.3)	0.54
LDL-c, mmol/l	2.3 ± 1.1	2.4 ± 1.1	0.23
HDL-c for female, mmol/l	1.08(0.92–1.26)	1.04(0.95–1.23)	0.50
HDL-c for male, mmol/l	0.93(0.82–1.09)	0.94(0.83–1.13)	0.98
ALT, u/l	20(14–31)	20(14–36)	0.35
AST, u/l	19(15–25)	20(15–26)	0.16
ALB, g/l	41.4 ± 4.1	41.4 ± 4.7	0.98
T-BIL, μmol/l	11.8(9.4–14.8)	13.2(10.4–17.9)	<0.001
CRE, μmol/l	64(53–75)	64(53–74)	0.76
UA for female, μmol/l	309(255–369)	319(272–372)	0.19
UA for male, μmol/l	361(314–416)	365(295–434)	0.72
UACR, mg/g	7.5(3.0–21.6)	8.3(3.7–25.4)	0.26
WBC, 10^9^/l	6.6 ± 1.7	6.7 ± 1.6	0.81
RBC, 10^12^/l	4.6 ± 0.5	4.6 ± 0.6	0.37
Hb, g/dl	14.0 ± 1.5	14.0 ± 1.6	0.76
PLT, 10^9^/dl	20.8 ± 5.5	20.7 ± 5.6	0.80
Smoking, n (%)	349(42.5)	88(30.1)	0.11
CVD, n (%)	98(11.9)	26(10.9)	0.66
CHD, n (%)	108(13.2)	29(12.2)	0.70
PAD, n (%)	653(79.5)	42(76.1)	0.19
DR, n (%)	170(20.7)	47(19.7)	0.73
DKD, n (%)	159(19.4)	43(18.1)	0.65
Hypertension, n (%)	406(49.5)	132(55.5)	0.07
Dyslipidemia, n (%)	602(73.3)	171(71.8)	0.57

Values are mean ± SD, median (interquartile range), or n (%).

WC, waist circumference; SBP, systolic blood pressure; DBP, diastolic blood pressure; ALB, serum albumin; T-BIL, total bilirubin; CRE, creatinine; UA, uric acid; TC, total cholesterol; TG, triglycerides; HDL-c, high-density lipoprotein-cholesterol; LDL-c, low-density lipoprotein-cholesterol; ALT, alanine transaminase; UACR, urine albumin-creatinine ratio; WBC, white blood cell count; RBC, red blood cell count; Hb, haemoglobin concentration; PLT, platelet count; CHD, coronary heart disease; PAD, peripheral arterial disease; CVD, cerebral vascular disease; DR, diabetic retinopathy; DKD, diabetic kidney disease.

## Discussion

In this study, we have found that STB is positively associated with haemoglobin concentration and serum albumin, and negatively with platelet count. However, serum albumin concentration, but not STB, was independently associated with DR and DKD. We have also replicated previous findings that the T allele of rs6742078 is independently associated with STB, but the prevalence of DR and DKD in patients with the T allele was similar to those without it, meaning that no association between DR or DKD and the T allele was identified.

Senescent or damaged red blood cells are degraded and release haem, which is broken down by haem oxygenase, generating biliverdin and carbon monoxide. Biliverdin reductase then reduces biliverdin to bilirubin. Through these mechanisms, cells are protected against haem accumulation. Bilirubin binds to plasma albumin, is transported to the liver and conjugated by UGT1A1^19^. Thus, it is not surprising that we found that STB is associated with haemoglobin concentration and red blood cell count, because these variables are directly related to the number of senescent red blood cells.

Because bilirubin binds to plasma albumin and is transported to the liver, and the concentration of free bilirubin in the plasma is normally very low (<0.01%)^20^, its concentration is substantially affected by the plasma albumin concentration. Hypertriglyceridaemia is a component of the metabolic syndrome characterised by central obesity, insulin resistance, chronic low-grade inflammation, hyperglycaemia, dyslipidaemia and hypertension^1–3^. In individuals with the metabolic syndrome, haem oxygenase is induced during oxidative stress and inflammation, which might lead to greater production of bilirubin^21^. Similarly, cigarette smoke can induce oxidative stress, with the same potential consequence^22^. However, once haem oxygenase is induced, the oxidative stress and inflammation should be ameliorated, which could help prevent platelet overactivity. Consistent with this, a patient with haem oxygenase-1 deficiency presented with thrombocytosis and lower bilirubin production^23^. Moreover, inhibition of haem oxygenase activity limits the production of bilirubin and enhances 15d-prostaglandin J2-induced platelet production^24^, which might explain why STB was negatively associated with platelet count.

Bilirubin is an antioxidant compound that has been demonstrated to inhibit lipid oxidation, the immune reaction, inflammation, cell proliferation and apoptosis, and thus many of the pathophysiological changes that are involved in the development of diabetes, diabetic vascular complications and cardiovascular disease^25^. In the past decade, a few cross-sectional studies, observational studies and meta-analyses have shown that people with low STB are at a higher risk of diabetes, DR, DKD, CVD and CHD^4–14^. However, in these previous studies, anaemia, low haemoglobin and low albumin were also reported to be associated with a higher risk of DR, DKD and cardiovascular disease^26–36^, and smoking and high platelet count are well-known risk factors. However, all the associations identified between STB and diabetic microvascular complications would have been lost if confounders such as haemoglobin and albumin had been adjusted for. Furthermore, we now show that STB is not associated with DR or DKD after these covariates are adjusted for. Because the liver has a remarkable ability for compensation and most patients with DKD are not macroalbuminuric, the associations between serum albumin and DR or DKD cannot be explained by the loss of albumin through the kidneys alone. Thus, we are confident that the observed associations between serum albumin and diabetic chronic vascular complications are real.

Albumin is synthesized in the liver and accounts for more than half of serum protein. Serum albumin has many physiological functions, including the transport of inorganic ions, fatty acids, bilirubin, hormones and drugs. In addition, it contains abundant thiol groups that can scavenge most of the reactive oxygen in the blood^37^. Low serum albumin concentration is associated with ischemic heart disease, heart failure, atrial fibrillation, stroke, venous thromboembolism, atherosclerosis, end-stage renal disease and inflammation, which is consistent with the antioxidant, anti-inflammatory, anticoagulant and anti-platelet aggregation activities of the protein^37,38^. Many factors can reduce albumin synthesis, including malnutrition, inflammation, diabetes, liver disease and sepsis; and greater catabolism, vascular permeability and renal or enteric loss can also lead to low serum albumin concentration^39^. It is likely that serum bilirubin bound to albumin only provides minor protection against oxidative stress, because the associations between STB and DR or DKD disappeared when serum albumin concentration was adjusted for in the present study. This implies that serum albumin might contribute to the development of diabetic chronic vascular complications through its antioxidant, anti-inflammatory and anticoagulant effects.

To identify a potential causal relationship between bilirubin and cardiovascular disease, several studies have been conducted using Mendelian randomization analysis^12–14^. In a Korean cohort of 4,793 healthy subjects and 806 stroke patients, both the rs6742078 SNP and the weighted genetic risk score at the *UGT1A1* locus were shown to be associated with STB, but no evidence was presented showing that serum bilirubin is causally associated with stroke risk^12^. Another large study of 67,068 individuals in three cohorts showed that STB is not causally associated with the risk of ischemic heart disease, within each cohort and in a meta-analysis^14^.

However, to date, a Mendelian randomization study has not been conducted of the relationships between STB and DR or DKD. In the present study, we genotyped 1,059 patients with T2DM and did not identify an association between rs6742078 and diabetic microvascular complications (DR or DKD) or the well-established risk factors for cardiovascular disease, implying no causal association of STB with DR or DKD. Instead, it may be that the associations between STB and chronic diabetic complications identified in previous studies might be due to greater activity of haem oxygenase, because the oxidative stress and inflammation present in patients with diabetes, which are related to serum albumin concentration, may activate haem oxygenase. Haem oxygenase protects cells by degrading haem and generating carbon monoxide, rather than the by-product of bilirubin^25,40^, and the association between the highly expressed allele of the haem oxygenase gene and low vascular disease risk^41^ supports the hypothesis that STB represents a marker of the production of bilirubin within tissues, rather than a key mediator of vascular disease.

In the present study, we have found that STB is not associated with DR or DKD after adjustment for confounders (haemoglobin concentration, platelet count and serum albumin) but that serum albumin is. These findings are consistent with the results of previous Mendelian randomization studies. Although there has been no previous Mendelian randomization study regarding the association of STB with chronic vascular complications, we hypothesise that bilirubin is not important in the development of DR or DKD because the pathogenesis of diabetic microvascular disease and microvascular disease of other aetiologies is similar, involving oxidative stress and inflammation. Mendelian randomization studies are useful for the identification of causal relationships between associated factors, and in the present study we found that a representative SNP in *UGT1A1* is not associated with diabetic vascular complications or the well-established risk factors for vascular disease, suggesting that bilirubin might not play an important role in the development of DR and DKD.

There were some limitations to this study. Firstly, the sample size of the genetic association study was not large enough to evaluate the relationship between an allele with a small effect and diabetic chronic vascular complications. The study described had enough power (>0.8) only for an allele with an odds ratio >1.46 in a dominant genetic model, when the prevalence of DKD was 0.2 in a T2DM population and the T allele frequency was 0.12 (http://zzz.bwh.harvard.edu/cgi-bin/cc2k.cgi). Therefore, a large Mendelian randomization study should be conducted in the future that involves multiple SNPs, to help clarify whether any causal relationship between STB and diabetic vascular complications exists. In addition, because many covariates were adjusted for in the multiple logistic regression (Table 2), a further study with a larger sample size is needed to confirm our findings. Secondly, because the study was cross-sectional in nature, conclusions regarding causality must be corroborated by future longitudinal studies. It is possible that serum albumin is merely a marker of oxidative stress and inflammation in diabetic patients. Thirdly, a total of 1,059 patients were genotyped for rs6742078 because DNA samples were not available from all the patients, which might have led to bias. Compared with the participants as a whole, this sub-sample contained higher percentages of women and older people, and people with higher SBP, TG and STB concentrations, lower LDL-c, HbA1c and shorter duration of diabetes (data not shown). However, these differences between the sub-sample and the full cohort may not have a large impact on the results of the association study of rs6742078 with STB, DR and DKD because the frequencies of the G and T alleles at rs6742078 were very similar to those stated for Chinese people in the 1,000-genome database (12% *vs*. 11%), the distribution of rs6742078 genotypes was in accordance with Hardy-Weinberg equilibrium, and except for STB, the other clinical features were similar between the people with GG and those with GT/TT genotypes.

In summary, serum bilirubin was associated with haemoglobin concentration, platelet count, serum albumin, triglycerides, duration of diabetes and smoking status in a Han Chinese population with T2DM. Serum albumin, but not bilirubin, was independently associated with DR and DKD. Thus, serum bilirubin may not contribute significantly to the development of diabetic chronic vascular complications.

## Data Availability

The data are available on request from the authors.
